# Cotton Coloration Using Holy Basil Leaves Extracts

**DOI:** 10.1021/acsomega.5c03204

**Published:** 2025-06-20

**Authors:** Tanvir Ahmed Chowdhury, Jahirul Islam Khandaker, Mohammad Abdul Gafur, Md. Reazuddin Repon, Alamgir Hossain, Md. Kamrul Islam, Md. Abdullah

**Affiliations:** † Department of Physics, 115523 Jahangirnagar University, Dhaka 1342, Bangladesh; ‡ Department of Textile Engineering, 130058 Daffodil International University, Dhaka 1216, Bangladesh; § Pilot Plant & Process Development Centre, 130051 Bangladesh Council of Scientific and Industrial Research, Dhaka 1205, Bangladesh; ∥ Department of Bioproducts and Biosystems, School of Chemical Engineering, 174277 Aalto University, Espoo 02150, Finland

## Abstract

In order to provide a sustainable substitute for synthetic dyes, this study investigates the environmentally friendly coloration of cotton fabric utilizing lemon juice as a biomordant and holy basil leaves extracts as a natural dye. Twelve samples of cotton single jersey fabric were mordanted with lemon juice and then dyed with holy basil leaves extracts under acidic, neutral, and alkaline conditions at 40, 60, 80, and 100 °C. The results showed that the fabric dyed in an alkaline medium at 100 °C had the best color uptake and the highest color strength (*K*/*S*), as well as the best fastness to rubbing, washing, perspiration, and light fastness properties. The scanning electron microscopy (SEM) test results empirically validate the dyeing process, as the highest surface scratches were observed on colored cotton fabric dyed in the alkaline medium at 100 °C. Thermal analysis (Tg, DTg, DTA) reveals that the dyed fabric has improved thermal stability, with delayed decomposition and reduced weight loss compared to the undyed fabric, indicating a protective interaction between the dye and cotton fibers. The Fourier-transform infrared (FTIR) spectroscopy analysis also confirmed the interaction of cotton fabric with lemon juice mordant and holy basil leaf extracted natural dye particles. X-ray diffraction (XRD) analysis demonstrates that the dyeing process preserves the crystalline cellulose structure while introducing less crystalline or amorphous components from the basil extract. This research promotes the potential of holy basil leaf extract and lemon juice as a natural dyeing system, particularly effective in an alkaline medium at 100 °C, promoting sustainable practices in textile coloration.

## Graphical abstract

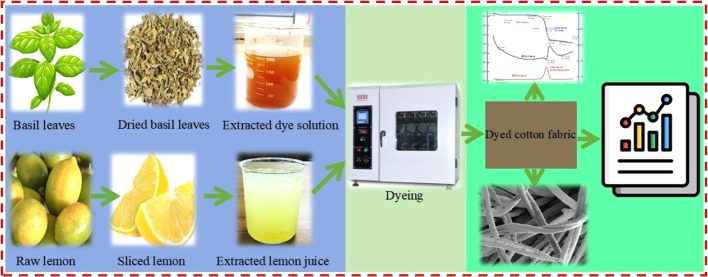

## 1 Introduction

Natural dyes (NDs) were utilized extensively prior to the introduction of synthetic dyes. Synthetic dyes (SDs) have been widely employed in the coloration of textile fibers due to their numerous advantages, including ease of application, cost-effectiveness, and moderate to excellent color fastness properties.^1,2^ However, these dyes have a nonbiodegradable nature, which makes them hazardous to the environment, and considering this issue, many countries have made restrictions on the application and manufacturing of azo dyes (−N=N−).^2–4^ Consumers are now compelled to use natural dyes due to strict regulations imposed by various international environmental organizations, such as the Food and Agriculture Organization (FAO), Environmental Protection Agency (EPA), Global Organic Textile Standard (GOTS), and Ecological and Toxicological Associations of Dyes and Pigments (ETAD),^5–7^ as well as growing health and environmental consciousness.^8–10^ Textile products made organically and posing no harm to the global population are supported by environmental groups.^5,11^ As a result, researchers, businessmen, and end users have begun to pay attention to the replacement of synthetic dyes in textiles with safer natural colors .^5,11,12^

Natural dyes are sustainable, have the potential to be renewable, and more environmentally friendly, biodegradable,^13^ and allergy-free^13,14^ than synthetic dyes. NDs also exhibit antibacterial, anti-inflammatory, antioxidant, anticancer, UV protective, insect repellent, and aromatic properties in textiles.^2,5,15–19^ Moreover, in recent years, there has been growing concern about the environmental impact of SDs. Additionally, the carcinogenicity of some diazo dyes has made SDs controversial and poorly accepted.^1^ The formation of toxic amines and visible remnants in effluents are the main sources of the difficulties of SDs.^20–23^ Hence, replacing SDs with NDs is a promising solution to reduce pollution and waste.^2^

Dyes extracted from natural resources like plants, animals, and minerals are known as natural dyes.^1,2,16,24,25^ However, plant dyes have pure dye concentration and better color production than SDs. Plants gain color from leaves, fruits, flowers, roots, and barks. The natural resources commonly used include orange, pomegranate, eucalyptus, kamala, madder, henna, and turmeric.^1,2^ Plant-based colors mostly contain flavonoids, anthraquinones, and indigo. Flavonols, flavanones, and anthocyanins are the most prevalent flavonoids. These flavonoids come in brown, yellow, and green colors.^24^ However, these dyes have technical constraints in terms of color fastness, low absorption,^26^ color production, repeatability, dyeing challenges, and mixing issues.

Poor color fastness is a common feature of NDs due to their limited affinity for textile fibers.^27^ Mordants are added to NDs to enhance their affinity with textile fibers during the dyeing process.^2,28,29^ A mordant increases dye absorption and retention, resulting in improved color fastness^21^ and a deeper shade.^29^ It also creates a chemical reaction and a bond between the dye and the fabric. Common mordants (metal salts) include ferrous sulfate, copper sulfate, chromium, alum, and stannous chloride. Mordant’s metal ions form covalent bonds with dye molecules.^2^ These mordents having heavy metal salts change a lot of natural colors.^21,27,29–32^ On the other hand, NDs have complex structures, and ions in metallic salts are water insoluble, which increases their fastness.^2,27,32^ When some metallic salts, such as copper and chromium, exceed a particular threshold, they are deemed undesirable heavy metals that are damaging to human skin.^29,33^ There are some plant-based natural resources used as natural mordants, e.g., lemon juice,^10,34^ tamarind, oxalic acid, citric acid, acacia catechu, etc.^1,10,35–37^

Plants produced around 2,000 color pigments, but only about 150 are commercially available. Many plant components, including roots, bark, stems, seeds, and fruit, contain natural dyes that may be extracted for color.^1^ Different parts of a plant can produce many colors.^38^ Tulsi (Basil), *Ocimum Sanctum* leaves produce green colors. These dyes are based on an anthraquinone structure, and plants that yield green dye are rare.^1^ The leaves of the *Ocimum tenuiflorum* (Black Tulsi, considered as holy basil in some countries) plant are commonly consumed in India due to their reputed antibacterial and therapeutic characteristics. Some researchers suggest that it may have anticancer, antidiabetic, analgesic, adaptogenic, and diaphoretic effects. Different constituents are present in different parts of the Tulsi plant. The main components of the leaf extract include triterpenes, flavonoids, and eugenol.^39–41^ Rosmarinic acid and ursolic acid are identified in the holy basil leaves, and these phenolic compounds are possibly responsible for pigmentation. Tulsi shows antibacterial and antimicrobial properties due to the presence of such chemical constituents.^42^ The chemical structure of major components present in holy basil leaf extracts is shown in Figure 1.

**Figure 1:**
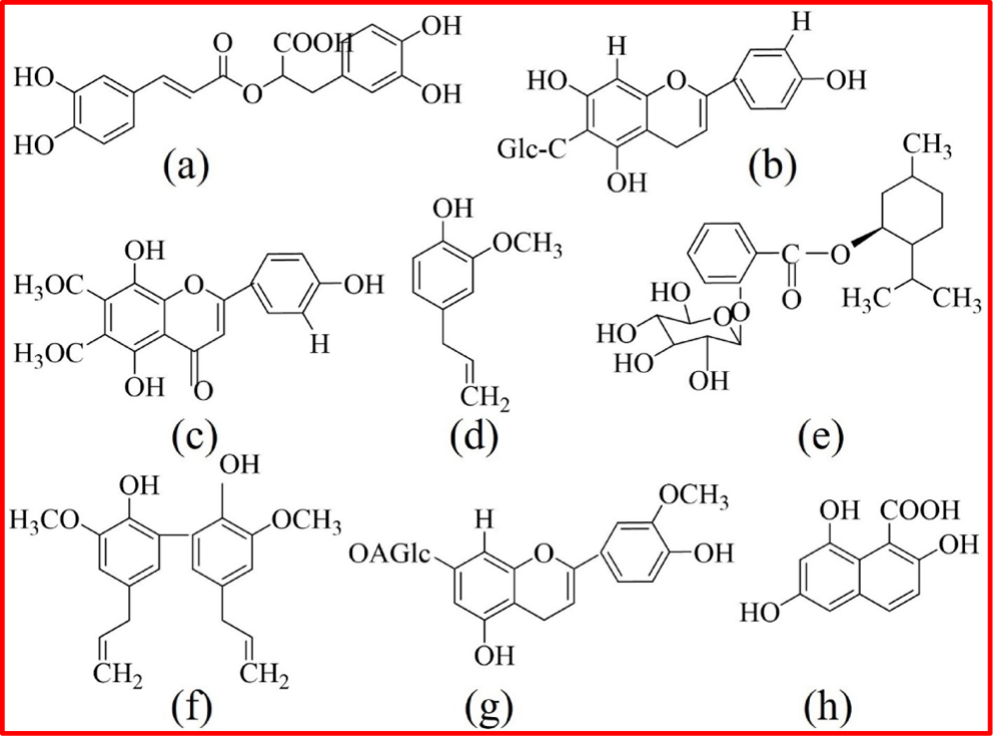
Chemical structure of major components present in holy basil leaves extracts: (a) Rosmarinic acid, (b) isovitexin, (c) isothymusin, (d) eugenol, (e) 1-menthyl-2-glucopyranosyloxybenzoate, (f) bieugenol, (g) crysoeriol, and (h) ocimumnaphthanoic acid.

Cotton is the most often used natural fiber in the worldwide textile industry due to its low cost, absorbency, breathability, and softness. It is resistant to natural colors, despite its strong affinity for SDs.^26^ Researchers have used many approaches to improve the natural dyeing of cellulosic fibers (e.g., cotton).^2,26,28,43^ Cotton dyeability has been enhanced through various methods, including enzymatic pretreatment, mordanting with metallic salts, premordanting,^2,44^ preparation with natural or synthetic tannins,^26^ plasma treatment,^45–47^ γ radiation,^48^ cationization,^49^ dyeing with ultrasonic energy, combining ozone and ultrasonic treatment,^50^ chitosan treatment,^51^ irradiation technologies,^16^ and acrylic acid grafting.^26^ In a recent study, artificial intelligence was used to forecast the link between the concentration of the specified components and each color coordinate separately.^52^ Various natural mordants and chitosan treatment have increased the dye absorption of cotton, and the fastness to washing, rubbing, and light has also been examined.^53,54^

A few studies were done using natural dye with biomordant. In fact, there is a lack of study on cotton knit fabric dyed with the *Ocimum tenuiflorum* (holy basil) leaves extract and lemon juice as biomordant to enhance color fastness. The measurement of reflectance (%), color strength, and color fastness of the dyed sample, as well as the chemical and physical changes of the dyed fabric in molecular state by XRD, FTIR, and SEM were used to characterize the dyed and undyed samples in this study.

## 2 Experimental Details

### 2.1 Materials

The pretreated 100% cotton knitted single jersey fabric having an areal density of 160 g per square meter, a wales per inch (WPI) of 35, courses per inch (CPI) of 53, yarn count 30 Ne, and a stitch length of 2.82 mm was fabricated at Wet Process Laboratory, Daffodil International University, Dhaka, Bangladesh. Holy basil (*Ocimum tenuiflorum*) leaves were used as the natural dye source, and lemon served as the biomordant in this study. The basil leaves were collected from tulsi plants, and the lemons were purchased from the Town Hall Market, Dhaka, Bangladesh. The pH of the dye bath was adjusted using acetic acid (CH_3_COOH) and sodium hydroxide (NaOH). A sulfonate-based soaping agent was applied during the postdyeing wash process.

### 2.2 Methods

#### 2.2.1 Dye Extraction Procedure

After collecting fresh basil leaves, they are dried under sunlight for 3 days until fully desiccated. The dried leaves are then ground into a fine powder. To extract the dye, 50 g of this powder is added to 1000 mL of water and heated to 100 °C, maintaining this temperature for 60 min. The mixture is then filtered to remove the leaf particles, resulting in a clear basil leaf extract. Figure 2a illustrates the basil leaf dye extraction procedure.

**Figure 2:**
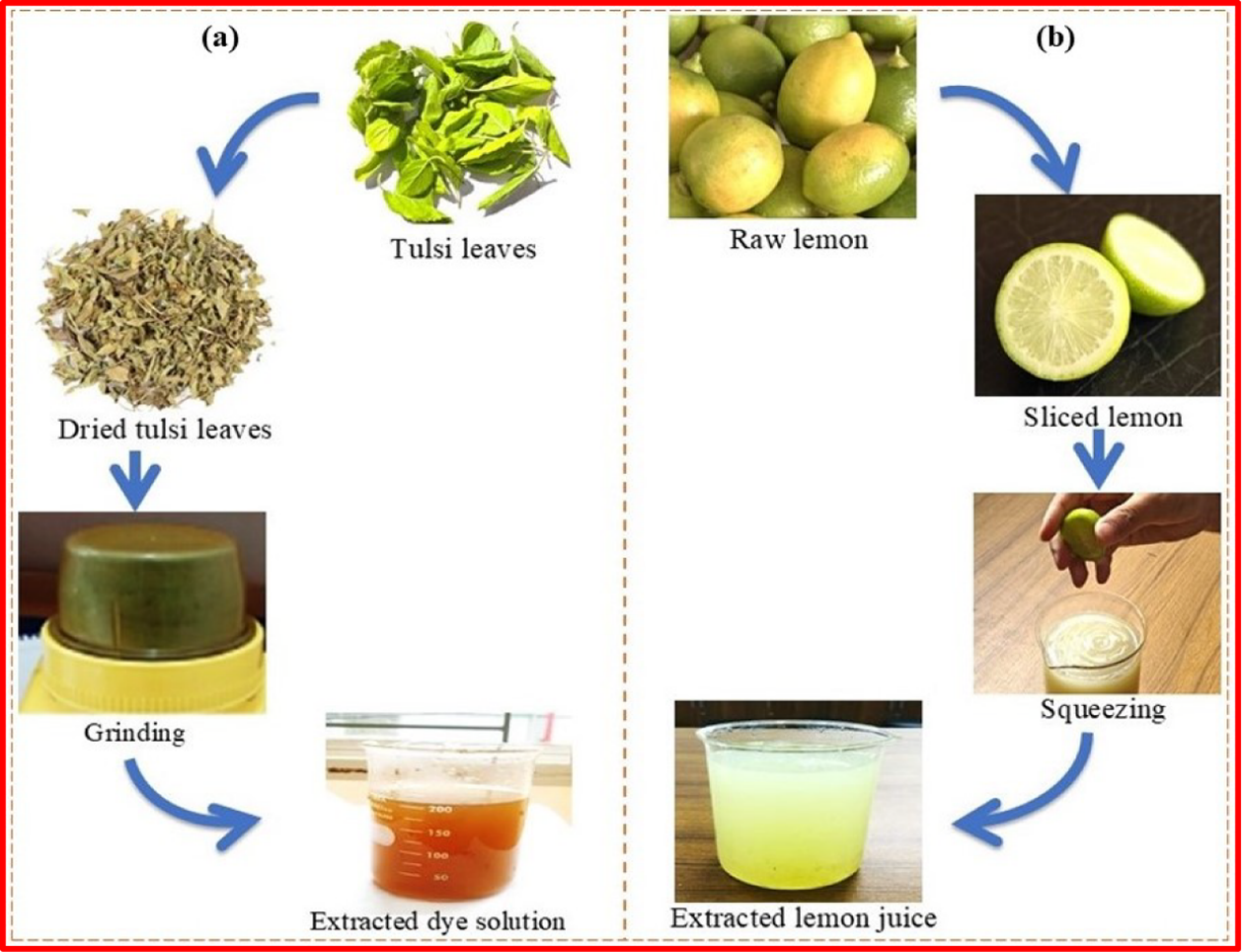
Extraction of dye from basil leaves (a) and lemon juice from lemon (b).

#### 2.2.2 Lemon Juice Extraction and Mordanting Procedure

Fresh lemons were cut into pieces, and the juice was extracted by using a suitable squeezer. The lemon juice was then filtered to remove any solids or unwanted particles. After filtration, 100 mL of lemon juice was mixed with 100 mL of water. A 5 g cotton fabric sample was immersed in this lemon juice solution for 24 h. Following the soaking period, the fabric was dried at 90 °C using a dryer for 3 min. Figure 2b illustrates the lemon juice extraction process.

#### 2.2.3 Dyeing Procedure

A 5 g mordanted fabric sample was immersed in 250 mL of the extracted dye solution. The pH levels (4.5, 7.0, and 11.0) were adjusted by adding acid or alkali. The samples were dyed at temperatures of 40, 60, 80, and 100 °C, with a dyeing time of 60 min in each case. After dyeing, the dye bath was drained, and the samples were washed with the soaping agent. This was followed by a hot wash at 80 °C for 3 min and a cold wash at room temperature (40 °C) for 3 min. Finally, the fabric was dried at 90 °C using a dryer for 3 min. The process curve is shown in Figure 3.

**Figure 3:**
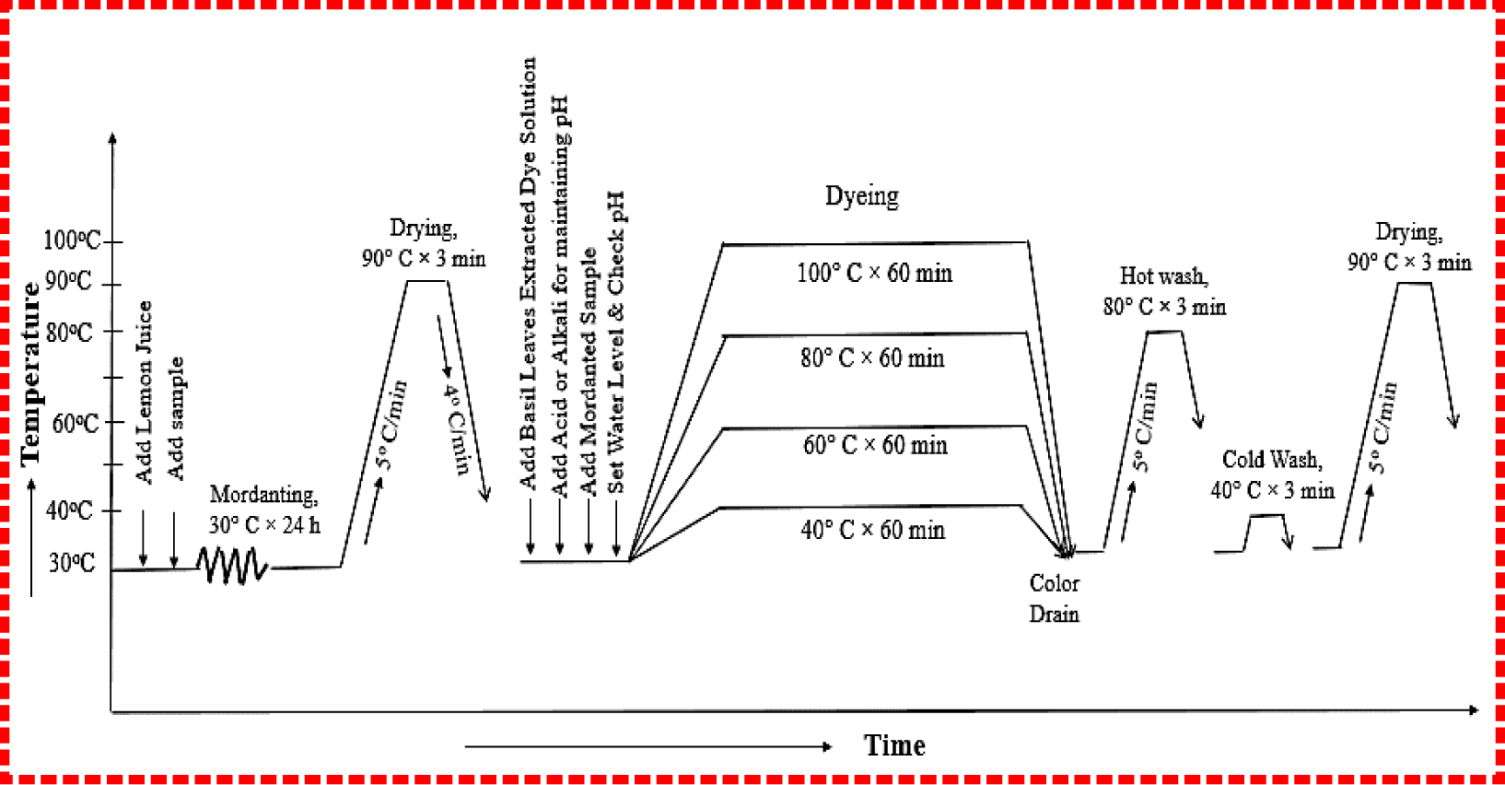
Process curve for mordanting, dyeing, and washing.

#### 2.2.4 Characterization

The color strength of the dyed cotton samples was determined using a Datacolor Spectro 1000, which measured the reflectance (%) values. The wavelength of maximum absorption (λmax) used for calculating the color strength (*K*/*S*) of the treated samples was 400 nm. The color strength values were calculated based on Kubelka–Munk’s theory, using the reflectance (%) data recorded from the samples.^55^ The *K*/*S* values were derived using eq 1.

1 $$\frac{K}{S}=\frac{\left(1-R\right)2}{2R}$$

where *R* is the reflectance, *K* is the absorption coefficient, and *S* is the scattering coefficient.

The color fastness to washing of the dyed samples was evaluated according to the ISO 105-C06:2010 standard method.^56^ Rubbing fastness was assessed using the ISO 105X-12:2016 standard,^57^ while perspiration fastness was tested following the ISO 105-E04:2013 method.^58^ In all cases, the results were rated using the gray scale. The color fastness to light was determined using the ISO 105-B01:2014 method,^59^ with ratings based on the blue wool scale.

Fourier transform infrared spectroscopy (FTIR) (Brand: PerkinElmer and Model: Spectrum Two, no. 123229) was utilized to measure spectral data of dyed and undyed fabric samples. Focus was placed on notable peaks within the fingerprint region (450–1800 cm^–1^) and functional group region (above 1800 cm^–1^) to characterize the differences between undyed and dyed samples.

Thermogravimetric analysis (Tg), derivative thermogravimetry (DTg), and differential thermal analysis (DTA) were performed to investigate the thermal stability of the dyed and undyed cotton fabrics. A thermogravimetric analyzer (TGA Q500) was used for the measurements under specific conditions.

In the X-ray diffraction (XRD) methodology, both dyed (using basil leaf extracted dye) and undyed cotton samples were prepared, dried, and mounted for analysis. Using an EMPYREAN diffractometer with a Cu anode and K-α1 radiation, scans were conducted from a start position of 2θ = 10.0234° to an end position of 2θ = 89.9734° to capture the peaks, with a scan step time of 7.14 s. Key data on peak intensities, positions, and amorphous content were collected to evaluate structural changes resulting from the dyeing process.

For scanning electron microscopy (SEM) analysis, undyed and dyed fabric samples were prepared by cleaning and cutting them into small sections, followed by coating them with a conductive layer to prevent charging. The samples were examined under high vacuum at magnifications of 100, 1000, and 2000× to capture surface morphology. This process highlighted structural changes caused by the interactions among the cotton fibers, lemon juice biomordant, and basil leaf extract.

## 3 Results and Discussion

### 3.1 CIELAB Color Coordinate Analysis

In order to investigate the CIELAB color coordinate analysis, the sample dyed at 40 °C and pH 7.0 was selected as the standard, representing the neutral condition where no acid or base was used. Eleven additional samples were treated as trial samples and dyed under various conditions to assess the influence of temperature and pH on dye uptake. Table 1 presents the CIELAB color coordinates, which indicate the color properties of each trial sample in comparison with the standard.

**Table 1:** Color Yield, Color Coordinates, and Color Difference of Dyed Fabrics^a^

					Color Coordinates						
Sample type	Min. *R*%	*K*/*S*	*K*/*S*%	Ill-obs	L*	a*	b*	c*	*h*°	ΔEcmc	MI
S-40, 7.0 (reference dyed)	8.54	4.90	100	D65–10	61.18	4.08	21.02	24.41	79.03	-	-
S-40, 4.5	8.14	5.18	105.7		–2.77	0.29	–0.61	–0.54	–0.41	1.28	0.09
S-40, 11.0	7.62	5.60	114.3		–3.42	0.01	–0.61	–0.60	–0.12	1.47	0.10
S-60, 7.0	9.37	4.38	89.2		2.00	0.03	–0.57	–0.56	–0.14	0.90	0.14
S-60, 4.5	7.79	5.46	111.4		4.67	–0.65	3.81	3.65	1.26	3.21	0.74
S-60, 11.0	7.02	6.16	125.0		2.25	–0.39	4.00	3.88	1.05	2.72	0.83
S-80, 7.0	8.71	4.78	97.6		3.14	–0.30	–0.19	–0.24	0.26	1.34	0.47
S-80, 4.5	7.80	5.45	111.2		–0.34	0.79	1.28	1.42	–0.51	1.02	0.25
S-80, 11.0	6.94	6.24	127.1		1.80	0.12	4.16	4.11	0.62	2.62	0.85
S-100, 7.0	7.88	5.38	109.8		–3.72	0.38	–1.66	–1.55	–0.72	1.96	0.11
S-100, 4.5	7.07	6.11	124.5		–5.74	0.35	–2.31	–2.19	–0.91	3.17	0.10
S-100, 11	6.40	6.84	139.2		–6.62	0.94	0.06	4.15	–0.83	2.59	0.44

a S = Sample, 40 = Temperature in °C, and 7.0 = pH.

The reference sample, S-40, 7.0 (dyed at 40 °C and pH 7), exhibited an L* value of 61.18, representing a relatively lighter shade. As the temperature increased and the pH shifted toward more acidic or alkaline conditions, the lightness values generally decreased, indicating the formation of darker shades. For example, Sample S-100, 11.0, dyed at 100 °C and pH 11.0, had the lowest L* value of −6.62, representing the darkest shade among the samples. The chroma (c*) values, which describe the intensity of the color, showed that the reference sample had the highest value (24.41), but the sample dyed at higher temperatures and pH values also exhibited high chroma. For instance, Sample S-100, 11.0, and c* values of 4.15, indicating a strong color intensity. The hue angle (*h*°) also shifted significantly with dyeing conditions. While the reference sample had a hue angle of 79.03°, Sample S-100, 11.0 displayed a much lower hue angle of −0.83°, indicating a pronounced shift toward cooler tones (blue/green). Furthermore, the color difference (ΔEcmc) values highlighted the variations in color compared with the reference sample. Sample S-60, 4.5 showed the highest ΔEcmc value of 3.21, indicating a significant deviation in color, while Sample S-60, 7.0 had the lowest ΔEcmc value of 0.90, meaning it closely resembled the reference sample. This suggests that dyeing at 60 °C and pH 7.0 produced the most consistent results with the reference.

The results show that higher temperatures and more extreme pH conditions (either acidic or alkaline) result in darker shades, higher color intensities, and greater variations in the reference sample. In particular, alkaline conditions at high temperatures (e.g., Sample S-100, 11.0) promoted the strongest dye uptake, as reflected by its high *K*/*S* value (6.84) and darker shade (L* = −6.62). These findings indicate that both temperature and pH are critical in optimizing the dyeing conditions for basil leaf dye.

### 3.2 Color Strength and Reflectance analysis

The analysis of color strength (*K*/*S*) and reflectance percentage of dyed cotton fabrics with basil leaves reveals significant variations based on dyeing conditions. As shown in Table 1, the reference sample, S-40, 7.0 (dyed at 40 °C and pH 7), yielded a *K*/*S* value of 4.90, establishing a baseline of 100% color strength. This indicates that under neutral conditions, the dye’s absorption is moderate. In contrast, the sample S-100, 11.0 (dyed at 100 °C and pH 11) exhibited a remarkable K/S value of 6.84 with the lowest reflectance of 6.40%, equivalent to 139.2% color strength, highlighting the effectiveness of alkaline conditions in enhancing dye uptake, likely due to improved dye-fiber interaction.

Furthermore, samples dyed at elevated temperatures and higher pH levels, such as S-60 at pH 11.0 (K/S 6.16) and S-80 at pH 11.0 (K/S 6.24), demonstrate that these conditions contribute to richer and deeper coloration. Conversely, S-60 at pH 7.0 displayed the lowest *K*/*S* value of 4.38, reflecting a higher reflectance of 9.37% (89.2% color strength), indicating that moderate dyeing conditions are less effective for achieving vibrant colors.

The data indicate that higher *K*/*S* values correlate with lower reflectance% (Figure 4), signifying increased color saturation. Additionally, both increasing pH levels and dyeing temperatures significantly enhance *K*/*S* values, highlighting the efficacy of alkaline conditions in promoting greater dye absorption and deeper, more vibrant colors on the fabric.

**Figure 4:**
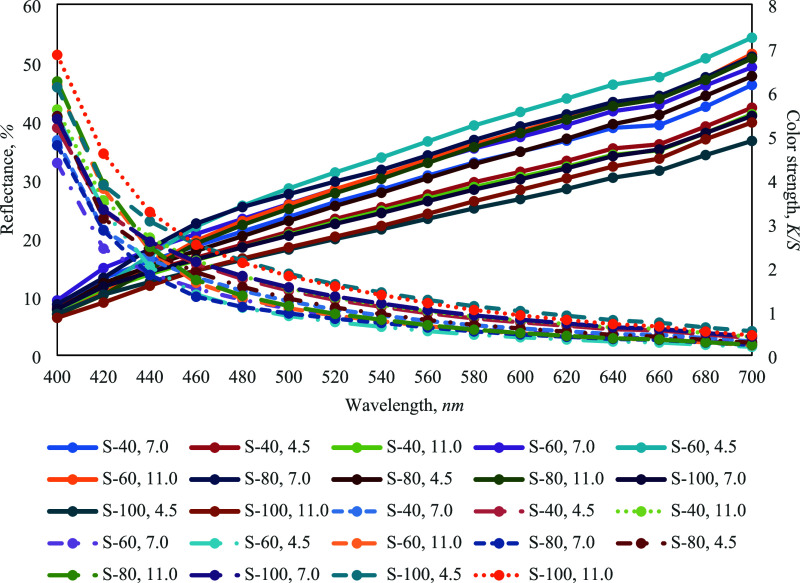
Reflectance% and color strength (*K*/*S*) of dyed samples.

### 3.3 Metamerism Index Analysis

Table 1 also represents the metamerism index (MI), a single value indicating the degree to which two samples that appear to match under one illuminant will maintain a match under another illuminant. The MI results were observed using the D65-10 light source. The analysis of the metamerism index (MI) for the dyed cotton fabric samples provides valuable insights into the color stability of the fabrics under different conditions of temperature and pH. The reference sample dyed at 40 °C and pH 7.0 serves as a baseline, exhibiting no metamerism and indicating consistent color perception across varying lighting conditions.

For the samples dyed at 40 °C, the MI values are relatively low, with pH 4.5 and 11.0 showing MI values of 0.09 and 0.10, respectively. The result suggests good color stability under these conditions, indicating that the color remains relatively consistent when viewed under different illuminants. As the temperature increases to 60 °C, the MI values begin to rise, indicating a potential increase in color inconsistency. The sample at pH 7.0 has an MI of 0.14, while the acidic condition (pH 4.5) shows a significantly higher MI of 0.74, reflecting a notable change in color perception. The alkaline condition (pH 11.0) at this temperature further exacerbates this issue, with an MI of 0.83, indicating considerable color variability. The trend continues with samples dyed at 80 °C, where the MI values remain high. The pH 7.0 sample shows an MI of 0.47, suggesting moderate metamerism, while the sample dyed at pH 11.0 reaches an MI of 0.85, indicating substantial color inconsistency. The sample dyed at pH 4.5 exhibits a lower MI of 0.25, suggesting better color stability than that of the alkaline counterparts. Finally, samples dyed at 100 °C present relatively low MI values for pH 4.5 and 7.0 (0.10 and 0.11, respectively), indicating good color consistency. However, the sample at pH 11.0 shows an MI of 0.44, suggesting some variability.

The results demonstrate that higher temperatures and alkaline pH levels (particularly pH 11.0) tend to increase the likelihood of metamerism in dyed fabrics. The significant variations observed highlight the importance of controlling both dyeing conditions and pH levels to minimize color inconsistencies and enhance color stability in practical applications.

### 3.4 Fastness Properties Analysis

The fastness properties of wash, rubbing, and perspiration fastness (Table 2) of the dyed cotton fabrics reveal insights into the durability and performance of the dye under various conditions. The wash fastness (color change) results across all samples exhibit consistent performance, with most samples showing grades of 3–4 or 4. The reference sample dyed at 40 °C, pH 7.0, achieves a wash fastness grade of 4, indicating good resistance to color change. Most other samples, including those dyed at 60, 80, and 100 °C, demonstrate similar levels of wash fastness, with only minor variations between grades 3–4 and 4, suggesting stable color retention during washing. Notably, samples dyed at more extreme conditions (e.g., S-100, 11.0) also exhibit strong wash fastness, maintaining a grade of 4.

**Table 2:**
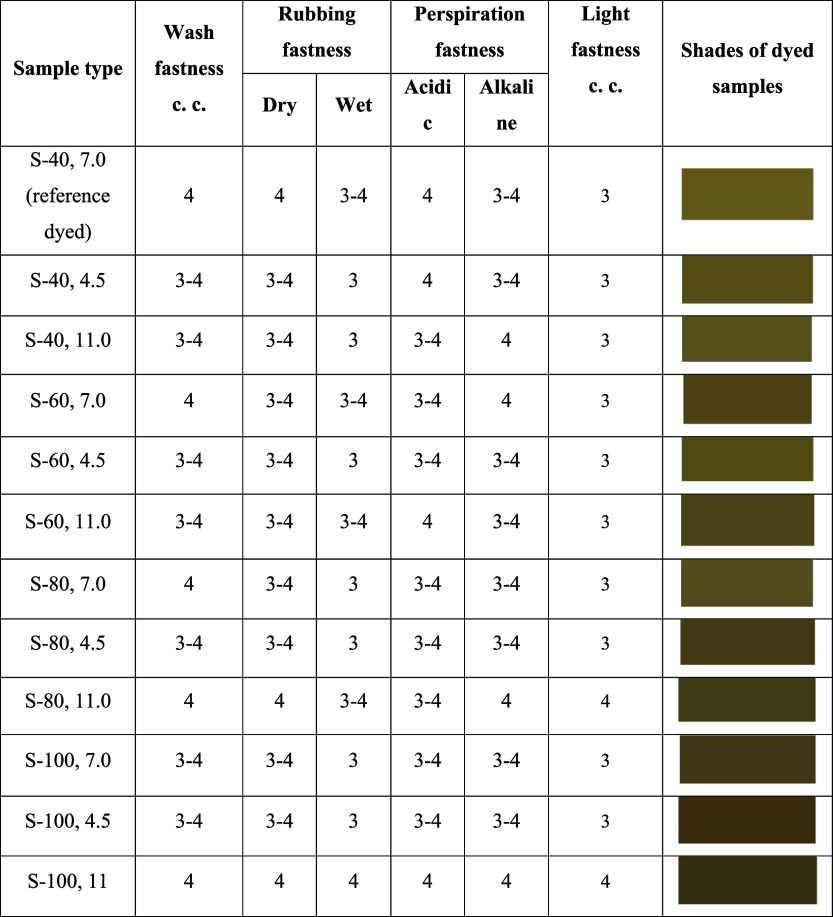
Color Fastness Properties of Dyed Samples^a^

a CC = Color change, S = Sample, 40, 60, 80, and 100 = Temperature in °C, and 4.5, 7.0, and 11.0 = pH.

In terms of rubbing speed, both dry and wet rub results are generally consistent across the samples. Dry rub fastness is relatively strong for most samples, with grades of 3–4 or 4 across the board. The reference sample (S-40, 7.0) scores 4 for dry rub, indicating excellent resistance to dry friction. Wet rub fastness, however, shows slightly lower performance compared to dry rub, with most samples scoring between 3 and 4. Samples dyed at higher temperatures and under alkaline conditions, such as S-100, 11.0, perform better with a wet rub fastness grade of 4, indicating stronger resistance to color loss during wet friction compared with lower pH or lower temperature conditions.

The perspiration fastness under acidic and alkaline conditions shows a similar performance for most samples, with grades ranging between 3 and 4. The reference sample dyed at 40 °C and pH 7.0 has grades of 4 under both acidic and alkaline perspiration, indicating strong resistance to perspiration-induced color changes. Samples dyed under more extreme conditions, such as at 100 °C with pH 11.0 (S-100, 11.0), exhibit the highest grades of 4 for both acidic and alkaline perspiration, indicating the dye’s robustness against perspiration. Other samples, especially those dyed at lower pH levels (e.g., S-40, 4.5 and S-100, 4.5), tend to score slightly lower with grades of 3 or 3–4, suggesting a moderate susceptibility to color change under perspiration.

Regarding light fastness, the samples generally show moderate resistance to color fading when exposed to light. The reference dyed sample (S-40, 7.0), along with most samples, received a light fastness grade of 3 on the Blue Wool Scale, which demonstrates below average performance. Samples S-80, 11.0 and S-100, 11.0 exhibit the highest light fastness grade of 4, highlighting comparatively better resistance to light-induced color fading. This improved performance can be attributed to the dyeing conditions: elevated temperatures (80 and 100 °C) combined with highly alkaline pH (11.0), which likely enhance dye penetration and fixation within the cotton fiber structure. Enhanced dye-fiber interactions under these optimum settings diminish the dye’s vulnerability to photodegradation, hence augmenting light fastness.

The fastness properties of the dyed fabrics indicate good durability under various conditions. The sample dyed at 100 °C with alkaline pH (11.0) showed superior fastness results. Higher dyeing temperatures and alkaline pH levels typically improve wash, rubbing, perspiration, and light fastness because these conditions promote better dye penetration and bonding to the fiber structure. At elevated temperatures, fiber swelling allows dyes to diffuse more easily into the fiber matrix, leading to stronger fixation.

### 3.5 FTIR Analysis of Dyed and Undyed Samples

Figure 5 shows that the Fourier-transform infrared (FTIR) spectra for both treated (with basil leaf extracted dye) and untreated cotton fabric samples were analyzed to understand the functional group changes induced by the dyeing process. Both untreated and dyed samples exhibit broad peaks around 3270–3350 cm^–1^, which are attributed to O–H stretching, typical of the cellulose in cotton fibers. The intensity of the O–H peak in the dyed sample shows a slight shift and increased sharpening, indicating hydrogen bonding interactions between the hydroxyl groups in the basil extract and the cellulose of the cotton fibers. For both samples, a peak near 2915 cm^–1^ is visible, linked to C–H stretching vibrations in alkanes, but it appears slightly more intense in the treated sample, suggesting some interaction between the plant compounds and the cotton fiber’s hydrocarbon structure.

**Figure 5:**
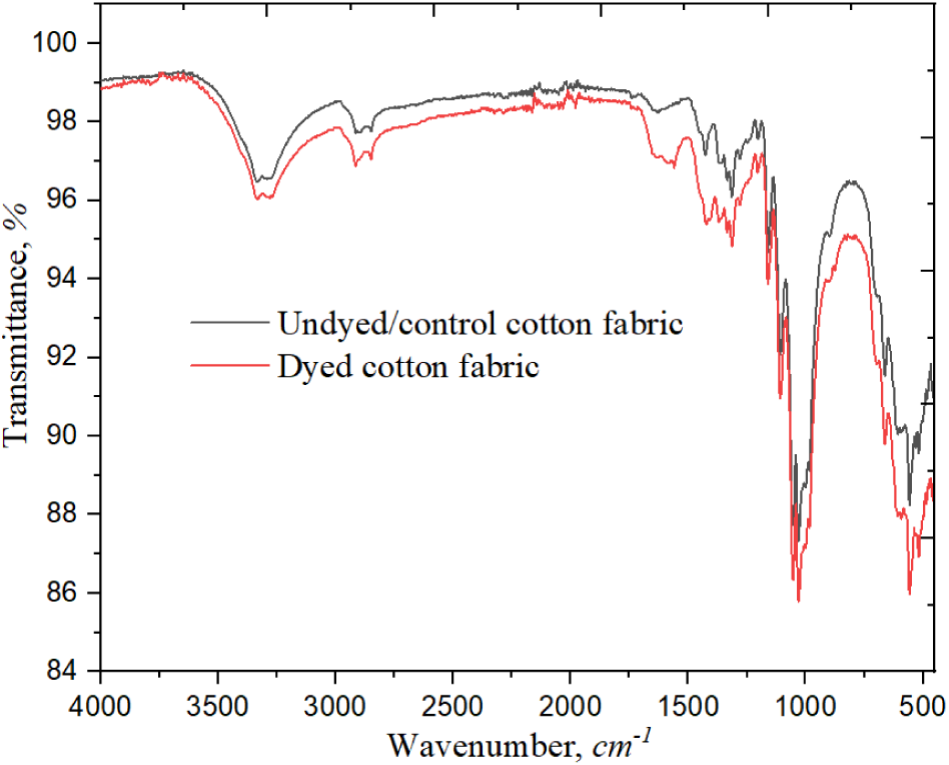
FTIR spectra of dyed and undyed cotton fabric.

In the fingerprint region, a peak observed in the dyed sample around 1633 cm^–1^ indicates carbonyl (C=O) stretching vibrations. This peak’s presence in the basil leaves extracted dyed sample and its relative absence in the untreated sample suggests the integration of carbonyl-containing compounds from the dye. This is likely from quinones or similar bioactive compounds within the basil extract, which may enhance dye adherence and colorfastness on cotton. The peak near 1450 cm^–1^ corresponds to aromatic C=C stretching, with a more pronounced peak appearing in the treated sample. This enhancement suggests an increased presence of phenolic compounds from the basil leaf extract, indicating successful integration of bioactive components onto the cotton fiber. Again, the peak between 1030 and 1130 cm^–1^ corresponds to C–O stretching, indicating alcohol or ether groups that could strengthen the interaction between the dye and the fiber. A distinct sharp peak near 550 cm^–1^ in the treated sample, less pronounced in the undyed sample, indicates increased aromatic ring deformations and confirms the successful integration of phenolic compounds from the basil leaf extract onto the cotton fiber. Together, these spectral variations indicate that the basil treatment effectively modifies the cotton fiber’s chemical structure, potentially providing functional benefits like enhanced stability and bioactivity, which are absent in untreated cotton.

### 3.6 Thermal Behavior Analysis

Thermal analysis techniques, such as thermogravimetric analysis (Tg), derivative thermogravimetry (DTg), and differential thermal analysis (DTA), were applied to both dyed and undyed samples.

The Tg curve shows the weight loss as a function of the temperature, which reflects the thermal stability of the material. The Tg curve for the undyed fabric sample (Figure 6) shows an initial weight loss of 5.1% up to 250 °C, followed by major decomposition starting at 350.1 °C. By 368.4 °C, the undyed fabric retains 47.9% of its initial weight, which continues to decrease until 534.3 °C, where approximately 6.3% of the weight remains. The Tg curve (Figure 6) for the dyed sample shows an initial weight loss of 3.2% up to about 200.8 °C, likely due to the evaporation of moisture and minor components. Major weight loss begins around 272.8 °C, with significant decomposition at around 318.1 °C, resulting in a 59.0% weight retention at this temperature. By 536.1 °C, the dyed sample has a residual weight of approximately 27.9%. The dyed fabric exhibits a slower decomposition rate in the initial phase, retaining a higher weight at lower temperatures. This indicates that the basil leaf extracted dye influences thermal stability, delaying complete decomposition compared to the undyed fabric.

**Figure 6:**
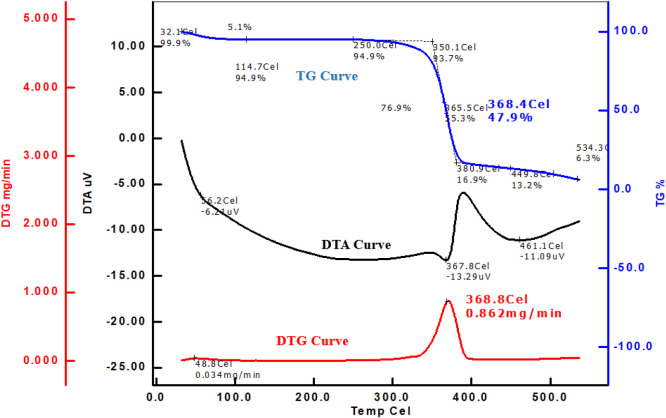
Tg, DTg, and DTA curves for the undyed fabric sample.

The DTg curve highlights the rate of weight loss and helps identify specific temperatures at which decomposition rates are highest. The DTg curve (Figure 7) for the dyed fabric shows a peak at around 321.1 °C with a maximum rate of weight loss of 0.392 mg/min. A secondary peak appears at 481.2 °C with a much lower rate (0.050 mg/min), indicating the final stages of decomposition. The undyed fabric has a more pronounced DTg peak at 368.8 °C with a maximum rate of weight loss of 0.862 mg/min. The lower peak intensity and shifted peak position in the DTG curve of the dyed fabric suggest an enhancement of thermal stability. This indicates that the dyeing process may have positively influenced the thermal properties of the cotton fabric, making it more resistant to thermal degradation compared to the undyed fabric.

**Figure 7:**
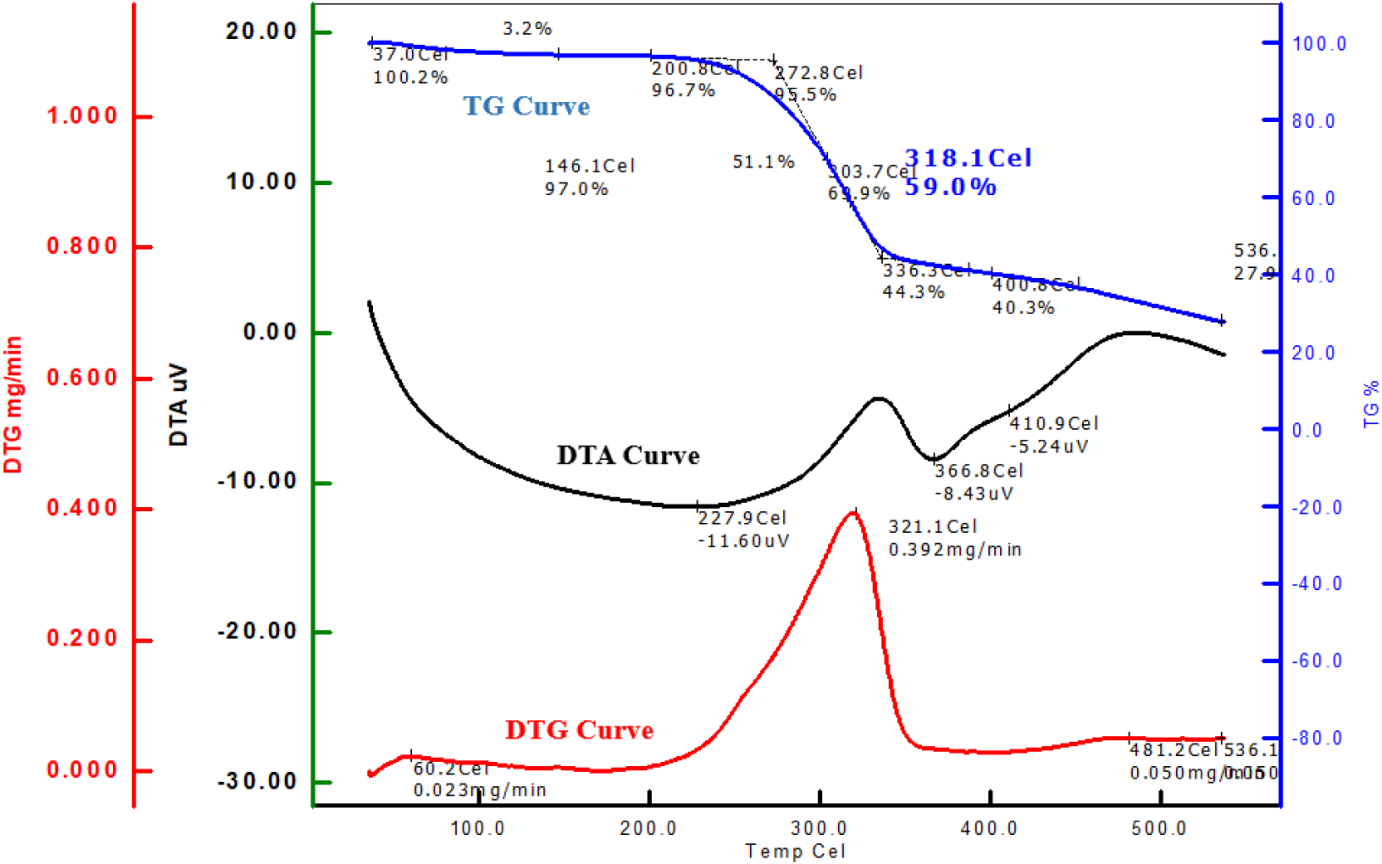
Tg, DTg, and DTA curves for the dyed fabric sample.

The DTA curve shows endothermic and exothermic transitions, providing further insight into phase changes and thermal reactions. The DTA curve for the undyed sample shows a more intense endothermic peak at 367.8 °C (−13.29 μV) and another peak at 461.1 °C (−11.09 μV), indicating a more rapid and intense thermal event associated with decomposition. The DTA curve for the dyed sample exhibits an endothermic peak at 227.9 °C (−11.60 μV), likely due to moisture loss or structural transitions within the fabric. A second endothermic peak appears around 410.9 °C (−5.24 μV), corresponding to a thermal degradation phase. The basil leaf extracted dyed fabric demonstrates a smoother DTA curve with less intense peaks, suggesting that the dye might stabilize the fabric, reducing the intensity of the exothermic or endothermic reactions. This stabilization effect indicates a slower thermal transition, likely due to the interaction of the dye with the cellulose structure.

The thermal analysis of basil leaf extracted dyed cotton fabric reveals improved thermal stability compared with undyed cotton. The dyed fabric shows delayed decomposition, lower weight loss rates, and moderate thermal transitions, which can be attributed to the protective interaction between the basil leaf extracted dye and cotton fibers.

### 3.7 XRD Analysis of Undyed and Dyed Samples

The X-ray diffraction (XRD) analysis of cotton fabrics, both undyed and dyed with basil leaf extracted dye, reveals valuable insights into the structural modifications of the fibers, as presented in Table 3 and Figure 8. For both samples, a prominent peak appears around 2θ = 21°, with the dyed sample showing a slightly higher intensity at this peak. This increased intensity suggests that the dyeing process may enhance the crystallinity or alignment of the cellulose chains, indicating that the fundamental crystalline structure of cellulose remains stable throughout the dyeing process. Moreover, the dyed fabric displays additional peaks within the 2θ range of 15–25° that are much less pronounced in the undyed sample. These peaks likely reflect minor crystalline or semicrystalline phases introduced by organic compounds within the basil extract, suggesting that while the core crystalline structure of cellulose is retained, new elements are incorporated into the fabric structure as a result of dyeing.

**Table 3:** Pattern List for Undyed and Dyed Samples

		Score				Scale Fac.		
Visible (both undyed and dyed)	Ref. Code (both undyed and dyed)	Undyed	Dyed	Compound Name (both undyed and dyed)	Displ. [°2θ] (both undyed and dyed)	Undyed	Dyed	Chem. Formula (both undyed and dyed)
*	00-050-2241	56	54	Cellulose	0.000	0.529	0.838	(C_6_H_10_O_5_)n

**Figure 8:**
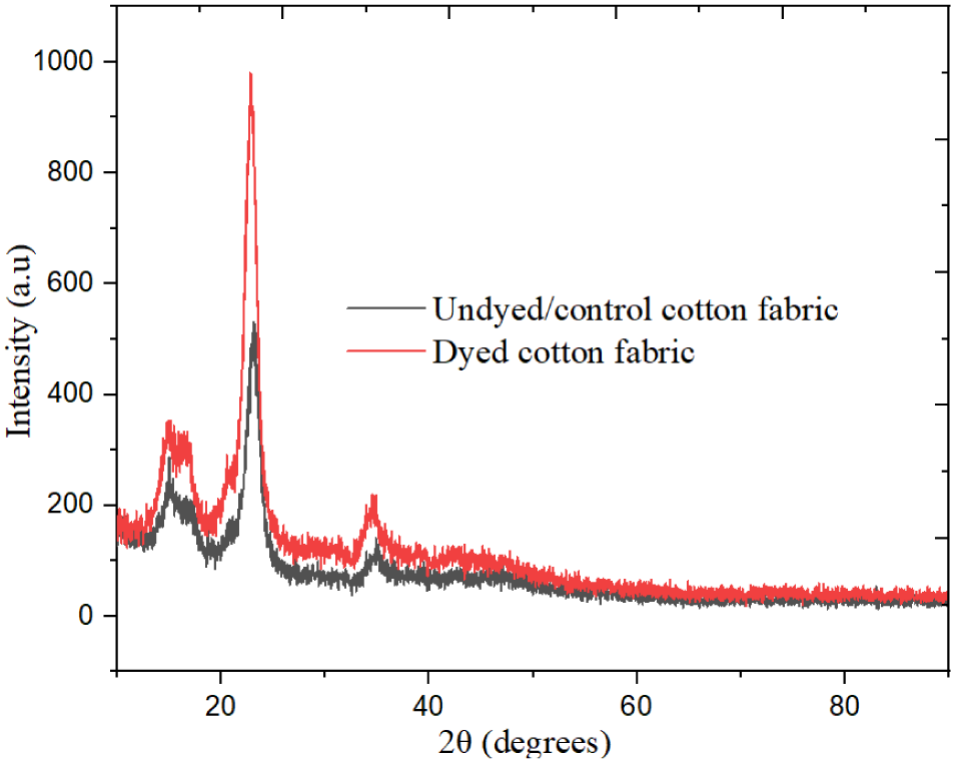
XRD of undyed and dyed samples.

The presence of cellulose is confirmed in both samples, validated by a pattern match with reference code 00-050-2241. The undyed sample recorded a score of 56, while the dyed sample showed a slightly lower score of 54, implying potential minor changes in the crystalline structure due to dyeing. Both samples also exhibit a broad amorphous background, characteristic of semicrystalline natural fibers such as cotton. This amorphous background is more pronounced in the dyed sample, which may point to an increase in amorphous content due to the deposition of noncrystalline organic compounds from the basil dye on the cotton fibers. Overall, these findings highlight the intricate interaction between basil dye and the cotton fibers. The dyeing process not only preserves the crystalline cellulose structure but also subtly modifies the fabric’s microstructure, introducing organic compounds that add to the complexity of the fiber composition.

### 3.8 Surface Morphology Analysis

Scanning electron microscopy (SEM) analysis was conducted to investigate morphological changes on the surfaces of the fabric samples. The SEM micrographs, captured at 100×, 1000×, and 2000× magnification, are depicted in Figure 9, illustrating both undyed and dyed cotton fabric.

**Figure 9:**
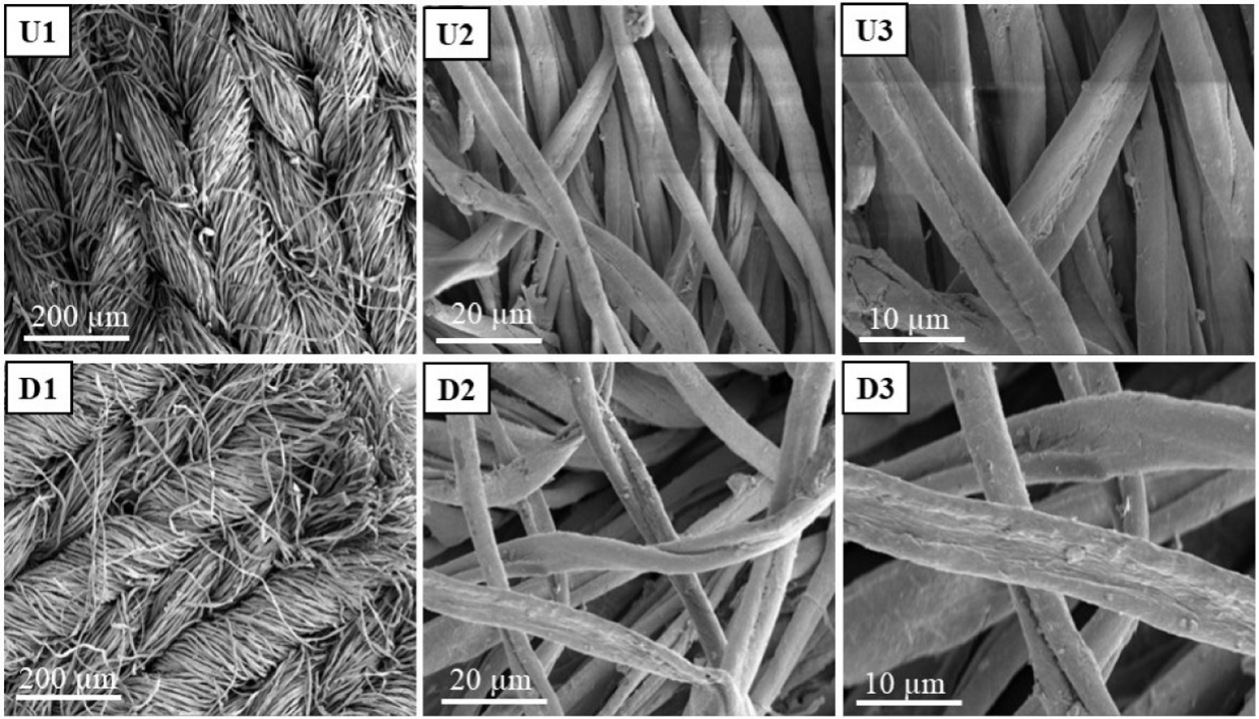
SEM images of undyed cotton fabric at different magnifications: 100× (U1), 1000× (U2), and 2000× (U3) and mordanted dyed cotton fabric at different magnifications: 100× (D1), 1000× (D2), and 2000× (D3).

Upon examination, SEM analysis of the control sample revealed a smooth fiber surface with no discernible physical modifications. In contrast, the SEM micrographs of mordanted dyed samples exhibited noticeable alterations in fiber surface morphology. The variance in color distribution in cotton fabric possibly stems from intermolecular forces at play among the cotton fabric, lemon juice, and the holy basil leaf dyestuff molecule. These findings provide empirical validation for the efficacy of the dyeing process for cotton fabric using a biomordant and natural dye sourced from lemon juice and holy basil, respectively.

## 4 Conclusion

In this study, cotton fabrics were dyed with naturally extracted holy basil dyes using lemon juice mordant at different pH and temperature media. Each sample is then subjected to a variety of tests, which produce a range of values and, in some cases, nearly identical results. Here, color strength (*K*/*S*) values are evaluated to characterize the surface color of the fabric, and it is shown that *K*/*S* values are higher when the samples are dyed under alkali condition at 100 °C. The dyed shades are also studied, and it was found that the best dyed shades are also obtained at alkali pH at 100 °C. By evaluating MI, it can be said that when the DEcmc values of dyed samples increased (when comparing with the standard sample), the MI values also increased. The *K*/*S* and reflectance curves concluded an inverse relationship between color reflectance and color absorption, with higher reflectance resulting in lower absorption and, thus, lower *K*/*S* values. Additionally, color fastness features such as wash fastness, rubbing fastness, perspiration, and light fastness were evaluated. The findings revealed that fabrics colored under alkaline conditions at 100 °C had improved fastness qualities in all tests. These studies demonstrate the efficacy of employing an alkaline pH and high temperatures to improve color intensity and durability in natural dyeing procedures.

FTIR study revealed the incorporation of bioactive chemicals from holy basil dye into cotton fabric, as evidenced by changes in functional groups such as hydroxyl (O–H), carbonyl (C=O), and aromatic C=C. This research confirmed the chemical interaction between the dye and fiber, revealing the presence of color components obtained from holy basil leaves. SEM imaging revealed a higher density of surface scratches on materials dyed under alkaline conditions at 100 °C, highlighting the dyeing process’s effectiveness. Thermal studies (Tg, DTg, and DTA) revealed that the dyed cloth was more thermally stable than its undyed counterpart with delayed decomposition and less weight loss. These findings establish the successful dyeing of cotton fabric utilizing holy basil extract and lemon juice as a biomordant. The X-ray diffraction (XRD) analysis demonstrated that the dyeing method preserved the cotton fabric’s crystalline cellulose structure while integrating less crystalline or amorphous components from the basil extract.

The findings showed that dyeing cotton fabric with holy basil extract and lemon juice as a biomordant in an alkaline solution at 100 °C produced better dyeing results than other examined samples. Moreover, this naturally dyed cotton fabric can be used for innerwear, babywear, sportswear, and other purposes where cotton fabric is used but light fastness is not important. This study reveals that using holy basil extract as a natural dye and lemon juice as a mordant offers a novel and environmentally beneficial way to textile dyeing. This research also expresses the promise of plant-derived colors and green mordants, which serve as sustainable substitutes for synthetic dyes in colored textile production to diminish production-related environmental damage. The current research requires expansion into enlarged applications and broader uses of natural dyes. Future investigations should direct their efforts toward increasing the industrial scale of natural dyeing to promote practical, real-world implementation. Research into optimizing natural dyeing processes, decreasing costs, and improving efficiency will transform natural dyeing into an acceptable option for commercial textile production

## Data Availability

All data presented herein are consistent with the published version.

## References

[1] Aggarwal S. (2021). Indian dye
yielding plants: Efforts and opportunities. Nat. Resour. Forum.

[2] Tayyab N., Sayed R. Y., Faisal R. (2020). Dyeing and colour fastness
of natural dye from Citrus aurantium on Lyocell fabric. Ind. Text..

[3] Islam T., Repon M. R., Islam T. (2023). Impact of textile dyes
on health and ecosystem: a review of structure, causes, and potential
solutions. Environ. Sci. Pollut. Res..

[4] Rahman M. R., James A., Mohamed Said K. A. (2024). A TiO 2 grafted bamboo
derivative nanocellulose polyvinylidene fluoride (PVDF) nanocomposite
membrane for wastewater treatment by a photocatalytic process. Mater. Adv..

[5] Adeel S., Zia K. M., Abdullah M. (2019). Ultrasonic assisted
improved extraction and dyeing of mordanted silk fabric using neem
bark as source of natural colourant. Nat. Prod.
Res..

[6] Parvinzadeh
Gashti M., Katozian B., Shaver M. (2014). Clay nanoadsorbent
as an environmentally friendly substitute for mordants in the natural
dyeing of carpet piles. Color. Technol..

[7] Kiumarsi A., Parvinzadeh Gashti M., Salehi P. (2017). Extraction of dyes from
Delphinium Zalil flowers and dyeing silk yarns. J. Text. Inst..

[8] Bechtold T., Turcanu A., Ganglberger E. (2003). Natural dyes in modern
textile dyehouses — how to combine experiences of two centuries
to meet the demands of the future?. J. Cleaner
Prod..

[9] Haddar W., Ben Ticha M., Guesmi A. (2014). A novel
approach for
a natural dyeing process of cotton fabric with Hibiscus mutabilis
(Gulzuba): Process development and optimization using statistical
analysis. J. Cleaner Prod..

[10] Chowdhury T. A., Khandaker J. I., Gafur M. A., Repon M. R., Islam M. K., Hossain A., Mollick S. (2025). Biomordant assisted natural dyeing
of cellulosic fibre: A greener approach. Mater.
Res. Innovations.

[11] Ghosh J., Repon M. R., Pranta A. D., Rupanty N. S., Khan F., Noor T. (2025). Bioactive component
integrated textiles: A promising source of medicine
and healthcare. J. Eng. Fibers Fabr..

[12] Zuber M., Adeel S., Rehman F.-U. (2020). Influence of Microwave
Radiation on Dyeing of Bio-mordanted Silk Fabric using Neem Bark (Azadirachta
indica)-Based Tannin Natural Dye. J. Nat. Fibers.

[13] Ebrahimi I., Parvinzadeh Gashti M. (2016). Extraction of polyphenolic dyes from henna, pomegranate
rind, and Pterocarya fraxinifolia for nylon 6 dyeing. Color. Technol..

[14] Adeel S., Zuber M., Fazal-Ur-Rehman (2018). Microwave-assisted extraction
and dyeing
of chemical and bio-mordanted cotton fabric using harmal seeds as
a source of natural dye. Environ. Sci. Pollut.
Res. Int..

[15] Chowdhury T. A., Khandaker J. I., Gafur M. A. (2025). Bio-colouration
of nylon
fabric using natural dyes and mordants. Mater.
Res. Innovations.

[16] Pizzicato B., Pacifico S., Cayuela D. (2023). Advancements
in Sustainable
Natural Dyes for Textile Applications: A Review. Molecules.

[17] Junita, Fauzi A. M., Sunarti T. C., Yulianto A. (2024). Prospects of The Development of The Sustainable Natural
Textile Dye Industry: A Systematic Literature Review. IOP Conf. Ser. Earth Environ. Sci..

[18] Che J., Yang X. (2022). A recent (2009–2021)
perspective on sustainable color and
textile coloration using natural plant resources. Heliyon.

[19] Ali
Khan M., Shahid-Ul-Islam, Mohammad F. (2016). Extraction of Natural Dye from Walnut Bark and its
Dyeing Properties on Wool Yarn. J. Nat. Fibers.

[20] Momotaz F., Repon M. R., Prapti U. S. (2025). Dyeing performance and
antimicrobial activity of cellulose-based biomaterials. Cellulose.

[21] Fröse A., Schmidtke K., Sukmann T. (2019). Application
of natural
dyes on diverse textile materials. Optik.

[22] Sarıkaya R., Selvi M., Erkoç F. (2012). Evaluation
of potential genotoxicity
of five food dyes using the somatic mutation and recombination test. Chemosphere.

[23] Yamjala K., Nainar M. S., Ramisetti N. R. (2016). Methods
for the analysis of azo dyes
employed in food industry–A review. Food
Chem..

[24] Ayele M., Tesfaye T., Alemu D. (2020). Natural dyeing of cotton
fabric with extracts from mango tree: A step towards sustainable dyeing. Sustainable Chem. Pharm..

[25] Repon M. R., Dev B., Rahman M. A. (2024). Textile dyeing using natural mordants
and dyes: A review. Environ. Chem. Lett..

[26] Haji A. (2017). Improved natural
dyeing of cotton by plasma treatment and chitosan coating. Optimization
by response surface methodology. Cellul. Chem.
Technol..

[27] Gawish S.
M., Mashaly H. M., Helmy H. M., Ramadan A. M., Farouk R. (2017). Effect of
Mordant on UV Protection and Antimicrobial Activity of Cotton, Wool,
Silk and Nylon Fabrics Dyed with Some Natural Dyes. J. Nanomed Nanotechnol..

[28] Buyukakinci Y. B., Karadag R., Guzel E. T. (2021). Organic cotton fabric dyed with dyer’s
oak and barberry dye by microwave irradiation and conventional methods. Ind. Text..

[29] Uddin M. G. (2014). Effects
of Different Mordants on Silk Fabric Dyed with Onion Outer Skin Extracts. J. Text..

[30] Shabbir M., Islam S. U., Bukhari M. N. (2017). Application of Terminalia
chebula natural dye on wool fiber—evaluation of color and fastness
properties. Text. Clothing Sustainability.

[31] Torgan E., Ozer L. M., Karadag R. (2015). Colorimetric
and fastness studies
and analysis by reversed-phase high-performance liquid chromatography
with diode-array detection of the dyeing of silk fabric with natural
dye Helichrysum arenarium. Color. Technol..

[32] Haar S., Schrader E., Gatewood B. M. (2013). Comparison
of Aluminum Mordants on
the Colorfastness of Natural Dyes on Cotton. Clothing Text. Res. J..

[33] Dweck A. C. (2002). Natural
ingredients for colouring and styling. Int.
J. Cosmet. Sci..

[34] Chowdhury T. A., Khandaker J. I., Gafur M. A. (2024). Bio- colouration of
nylon fabric using natural dyes and mordants. Mater. Res. Innovations.

[35] Hossain A., Islam A. S., Samanta A. K. (2018). Pollution
Free Dyeing on Cotton Fabric
Extracted from Swietenia macrophylla and Musa Acuminata as Unpolluted
Dyes and Citrus. Limon (L.) as Unpolluted Mordanting Agent. Trends Text. Eng. Fashion Technol..

[36] Lohtander T., Arola S., Laaksonen P. (2020). Biomordanting
willow bark dye on
cellulosic materials. Color. Technol..

[37] Yusuf M., Mohammad F., Shabbir M. (2017). Eco-dyeing of wool with
Rubia cordifolia root extract: Assessment of the effect of Acacia
catechu as biomordant on color and fastness properties. Text. Cloth Sustainability.

[38] Sinha K., Saha P. D., Datta S. (2012). Response surface optimization
and
artificial neural network modeling of microwave assisted natural dye
extraction from pomegranate rind. Ind. Crops
Prod..

[39] Banerjee P., Sau S., Das P., Mukhopadhyay A. (2014). Green Synthesis
of Silver - Nanocomposite
for Treatment of Textile Dye. Nanosci. Technol.
Open Access.

[40] Almatroodi S. A., Alsahli M. A., Almatroudi A. (2020). Ocimum sanctum: Role
in Diseases Management Through Modulating Various Biological Activity. Pharmacogn. J..

[41] Rohit J. K. (2024). Eco-Friendly
Antimicrobial and UV Protection Functional Finishing of Cotton and
Bamboo Fabric using Tulsi (Ocimum sanctum). Int. J. Sci. Res..

[42] Singh D., Chaudhuri P. K. (2018). A review on phytochemical and pharmacological properties
of Holy basil (Ocimum sanctum L.). Ind. Crops
Prod..

[43] Manyim S., Kiprop A. K., Mwasiagi J. I. (2022). Dyeing
of cotton fabric
with Euclea divinorum extract using response surface optimization
method. Res. J. Text. Apparel.

[44] Hosseinnezhad M., Gharanjig K., Adeel S., Mahmoudi Nahavandi A. (2025). Introduction
of new combination of bio-mordant from agriculture waste for eco-dyeing
of wool yarns. Res. J. Text. Apparel.

[45] Gorjanc M., Savić A., Topalić-Trivunović L. (2016). Dyeing of plasma treated
cotton and bamboo rayon with Fallopia japonica
extract. Cellulose.

[46] Grujić D., Savić A., Topalić-Trivunović L. (2015). The influence of plasma
pretreatment on the structure and antimicrobial
properties of knitted fabrics treated with herbal extracts. ACC J..

[47] Haji A., Naebe M. (2020). Cleaner dyeing of textiles using
plasma treatment and natural dyes:
A review. J. Cleaner Prod..

[48] Batool F., Adeel S., Azeem M. (2013). Gamma radiations induced
improvement in dyeing properties and colorfastness of cotton fabrics
dyed with chicken gizzard leaves extracts. Radiat.
Phys. Chem..

[49] Fu S., Hinks D., Hauser P. (2013). High efficiency ultra-deep
dyeing of cotton via mercerization and cationization. Cellulose.

[50] Benli H., Bahtiyari M. İ. (2015). Combination of ozone and ultrasound in pretreatment
of cotton fabrics prior to natural dyeing. J.
Cleaner Prod..

[51] Morakotjinda P., Nitayaphat W. (2015). Dyeing Properties
and Color Fastness of Chitosan Treated
Cotton Fabrics with Thian King Leaves Extract. Appl. Mech. Mater..

[52] Haji A., Vadood M. (2023). Prediction of Color
Coordinates of Cotton Fabric Dyed
with Binary Mixtures of Madder and Weld Natural Dyes Using Artificial
Intelligence. Fibers Polym..

[53] Hwang E.-K., Lee Y.-H., Kim H.-D. (2008). Dyeing,
fastness, and deodorizing
properties of cotton, silk, and wool fabrics dyed with gardenia, coffee
sludge, Cassia tora. L., and pomegranate extracts. Fibers Polym..

[54] Lee Y.-H., Hwang E.-K., Kim H.-D. (2009). Colorimetric Assay
and Antibacterial
Activity of Cotton, Silk, and Wool Fabrics Dyed with Peony, Pomegranate,
Clove, Coptis chinenis and Gallnut Extracts. Materials.

[55] Alcaraz
de la Osa R., Iparragirre I., Ortiz D., Saiz J. M. (2020). The extended
Kubelka–Munk theory and its application to spectroscopy. ChemTexts.

[56] ISO. Tests for colour fastnessPart E04: Colour fastness to perspiration;(ISO 105-E04). 2013. https://www.iso.org/standard/57973.html.

[57] ISO. Tests for colour fastnessPart X12: Colour fastness to rubbing;(ISO 105-X12). 2016. https://www.iso.org/standard/65207.html.

[58] ISO. Tests for colour fastnessPart E04: Colour fastness to perspiration;(ISO 105-E04:). 2013. https://www.iso.org/standard/57973.html.

[59] ISO. Tests for colour fastnessPart B01: colour fastness to light: daylight(ISO 105-B01). 2014. https://www.iso.org/standard/65210.html

